# Environmental filtering increases with elevation for the assembly of gut microbiota in wild pikas

**DOI:** 10.1111/1751-7915.13450

**Published:** 2019-06-10

**Authors:** Huan Li, Rui Zhou, Jianxiao Zhu, Xiaodan Huang, Jiapeng Qu

**Affiliations:** ^1^ School of Public Health Lanzhou University Lanzhou 730000 China; ^2^ Key Laboratory of Restoration Ecology of Cold Area in Qinghai Province Xining Qinghai 810008 China; ^3^ State Key Laboratory of Grassland Agro‐ecosystems College of Pastoral Agriculture Science and Technology Lanzhou University Lanzhou 730020 China; ^4^ Key Laboratory of Adaptation and Evolution of Plateau Biota Northwest Institute of Plateau Biology Chinese Academy of Sciences Xining Qinghai 810008 China

## Abstract

Despite their important roles in host nutrition, metabolism and adaptability, the knowledge on how the mammalian gut microbial community assemble is relatively scanty, especially regarding the ecological mechanisms that govern microbiota along environmental gradients. To address this, we surveyed the diversity, function and ecological processes of gut microbiota in the wild plateau pika, *Ochotona curzoniae*, along the elevational gradient from 3106 to 4331 m on ‘the Roof of the World’—Qinghai‐Tibet Plateau. The results indicated that the alpha, beta and functional diversity of gut microbiota significantly increased with elevation, and elevation significantly explained the variations in the gut microbial communities, even after controlling for geographical distance, host sex and body weight. Some gene functions (e.g. nitrogen metabolism and protein kinases) associated with metabolism were enriched in the high‐altitude pikas. Null model and phylogenetic analysis suggest that the relative contributions of environmental filtering responsible for local gut communities increased with elevation. In addition, deterministic processes dominated gut microbial communities in the high‐altitude (more than 3694 m) pikas, while the percentages of stochastic and deterministic processes were very close in the low‐altitude (3106 and 3580 m) pikas. The observed mechanisms that influence pika gut microbiota assembly and function seemed to be mainly mediated by the internal gut environment and by the external environmental pressure (i.e. lower temperature) in the harsh high‐altitude environment. These findings enhance our understanding of gut microbiota assembly patterns and function in wild mammals from extreme harsh environments.

## Introduction

The microbes that inhabit on and inside humans and animals have aroused the interests of  scientists all over the world, not only because of their complexity and diversity, but also because they possess a lot of important functions on host, including food digestion (Tremaroli and Bäckhed, 2012), immunity regulation (Round and Mazmanian, 2009), disease prevention (Tlaskalova‐Hogenova *et al*., 2011) and physical development (Sommer and Backhed, 2013). The processes that influence diversity, functions and structures of these microbial communities are not adequately understood although we are eager to manipulate them to restore and maintain human health. In recent years, the application and development of high‐throughput sequencing techniques allow us to investigate and describe the composition and diversity of microbial communities with unprecedented depth, whereas the knowledge on how they assemble remains insufficient. To address this question, one effective approach is to borrow a conceptual framework from macro‐ecology, in which human and animal microbiota may be regarded as ecological communities and their hosts are viewed as ecosystems (Costello *et al*., 2012; Burns *et al*., 2015; Yan *et al*., 2016). This method is enormously attractive because it allows us to explore and understand how complex microbial communities assemble using macroscopic ecology methods.

In host–microbe systems, there are many factors (i.e. host genetics, development, diseases and diet) that influence the community assembly (Burns *et al*., 2015; Carmody *et al*., 2015; Rothschild *et al*., 2018). From a broad angle, community assembly is mediated by the three major categories of factors: (i) host traits, such as host species, genotype and immunity (Ley *et al*., 2008; Round and Mazmanian, 2009; Benson *et al*., 2010); (ii) microbe–microbe interactions, such as competitive and mutualistic interactions among species (Levy and Borenstein, 2013; de Muinck *et al*., 2013; Coyte *et al*., 2015); (iii) environmental factors, such as diet (Carmody *et al*., 2015), temperature (Kohl and Yahn, 2016) and elevation (Zhang *et al*., 2018). Although there are many potential factors that may contribute to community assembly in ecology, the ecological processes responsible for community assembly can be divided into two major categories, including (i) stochastic processes, in which species are independent of respective niches in habitats. The community assembly is influenced by stochastic events, such as stochastic dispersal (randomly sampling species from a regional species reservoir), colonization (accidental colonization of species), death (unpredictable death of species) and ecological drift (the stochastic loss and replacement of species). Thus, site–site variations (including alpha and beta diversity) of communities are generally undirectional and unpredictable (Moral and Lacher, 2005; Costello *et al*., 2012; Li *et al*., 2016a); (ii) deterministic processes, in which the establishment and boom of species in an environment largely rely on their respective niches and ecological fitness. In this case, community assembly is mediated by deterministic factors, such as abiotic (e.g. pH, resource availabilities) (Drury and Nisbet, 1973; Tripathi *et al*., 2018) and biotic factors (e.g. competitive or mutualism interactions among species) (Huston and Smith, 1987). Hence, the community diversity and structure can be directional and predictable (Anderson, 2007; Li *et al*., 2016a). Although the knowledge on microbial community assembly in several ecosystems (e.g. gut, soil, water) increases in recent years (Zhou *et al*., 2014; Yan *et al*., 2016; Tripathi *et al*., 2018), the debates on different ecosystems are dominated by stochastic or deterministic processes still continue. For example, gut microbiota assembly of the fruit fly (*Drosophila melanogaster*) was mainly governed by stochastic processes (Adair *et al*., 2018), while most of fish (including *Ctenopharyngodon idellus*,* Siniperca chuatsi* and *Silurus meridionalis*) gut communities were dominated by deterministic processes (Yan *et al*., 2016). This implies that the relative contributions of stochastic versus deterministic process are distinct in different ecosystems. Even the relative importance of these processes can vary in a specific ecosystem as time or environment changes (Tripathi *et al*., 2018). The assembly of gut microbiota should be more complicated than that of non‐host environmental microbiota in other systems (i.e. soil and water), because gut microbial communities are not only influenced by host traits, but also mediated by environments (Benson *et al*., 2010). Notably, one recent study found that environments probably play more important roles than host genetics in shaping gut microbiota (Rothschild *et al*., 2018). Thus, exploring the changes of gut microbiota assembly along environmental gradients has significant implications for manipulation of health microbiota by regulating environmental factors.

Plateau pika (*Ochotona curzoniae*), a member of the Ochotonidae family, is an important high‐altitude model animal and broadly distributed in the 3000–6000 m above sea level (ASL) on the Qinghai‐Tibet Plateau (Luo *et al*., 2008). Plateau pika is also an ancient native species, as some pika fossils found on the Qinghai‐Tibet Plateau are identified as approximately 20–30 million years old (Wang *et al*., 2008). Importantly, plateau pika plays a key role in alpine ecosystem function, including increasing plant diversity through creating micro‐habitats (burrows), providing food resources for carnivorous animals, improving soil structure, permeability and mixing, and promoting the circulation of ecosystem substances (Smith and Foggin, 1999; Li *et al*., 2013). These unique features make it especially attractive for studying the gut microbial community assembly along environmental gradients. First, because high altitude is positively associated with environmental stress, such as low pressure, low oxygen, cold climate and high ultraviolet radiation (Beall, 2006; Jablonski and Chaplin, 2010), the ecological processes responsible for gut microbiota assembly possibly exhibit different patterns compared with those low‐altitude animals. Second, through a long evolutionary process, plateau pikas have well adapted to the high‐altitude environments by the adaptation of host genes (Yang *et al*., 2007; Luo *et al*., 2008) and physiology (Du and Li, 1982; Du *et al*., 1984; Ge *et al*., 1998), whereas the adaptation of microbial communities for high altitudes is still elusive, as gut microbiota is associated with host nutrition and metabolism (Tremaroli and Bäckhed, 2012). Third, pikas are mammals and their gut is generally considered as sterile at birth, and thus, the gut may acquire microbes through horizontal (i.e. get colonists from environment) and vertical (i.e. inherit microbes from the mother) transmission in each new generation (Li *et al*., 2016b). Pika gut microbiota composition is influenced both by host traits and environment. This makes it more complex for exploring the microbial community assembly compared with non‐host environmental microbiota. Finally, the knowledge on pika gut microbiota assembly also can be extended to other mammals, including humans, because their digestive systems are very similar in many aspects (Langer, 2002).

The present study aims to reveal the ecological processes responsible for gut microbiota assembly in the ‘ecosystem engineers’—plateau pikas along altitudinal gradients on the Qinghai‐Tibet Plateau. Our previous studies and some other reports on pika gut microbiota suggested that host (e.g. genetics, population density, gut regions) (Li *et al*., 2016c, 2017a, 2017b) and environmental factors (e.g. diet, environmental microbes) (Li *et al*., 2016b, 2016d; Kohl *et al*., 2018) considerably influence the gut community composition and diversity patterns. Thus, the relative importance of stochastic versus deterministic processes that govern gut community assembly may be dynamic and changeable in different elevations. In this case, we address five key questions: (i) Which microbial taxa are associated with elevation? (ii) Does elevation predict alpha and beta diversity of pika gut microbiota? (iii) Which gene functions are enriched in high‐altitude pikas? (iv) Is the assembly of pika gut microbiota mainly governed by stochastic or deterministic processes? (v) Are the environmental filtering  positively correlated with elevation due to the increasing environmental stress in the high‐altitude regions? Our results are of significances for studying the gut microbiota assembly and function in wild mammals for extreme high‐altitude environments.

## Results

### The relationship between specific gut bacterial taxa and elevation

A total of 2, 511, 666 sequences produced based on MiSeq sequencing. After data standardization, we detected a total of 32,197 operational taxonomic units (OTUs) from 85 pika individuals (Uclust, 97% cut‐off). The dominant phyla (mean relative abundance > 1%) across all samples in the pika gut included *Firmicutes* (45.49%), *Bacteroidetes* (38.06%), *Proteobacteria* (5.68%) and *Spirochaetes* (2.54%). At genus level, five most abundant identified genera were *Prevotella* (11.64%), *Oscillospira* (7.27%), *Ruminococcus* (2.30%), *Treponema* (2.07%) and *YRC22* (1.24%).

**Figure 1:**
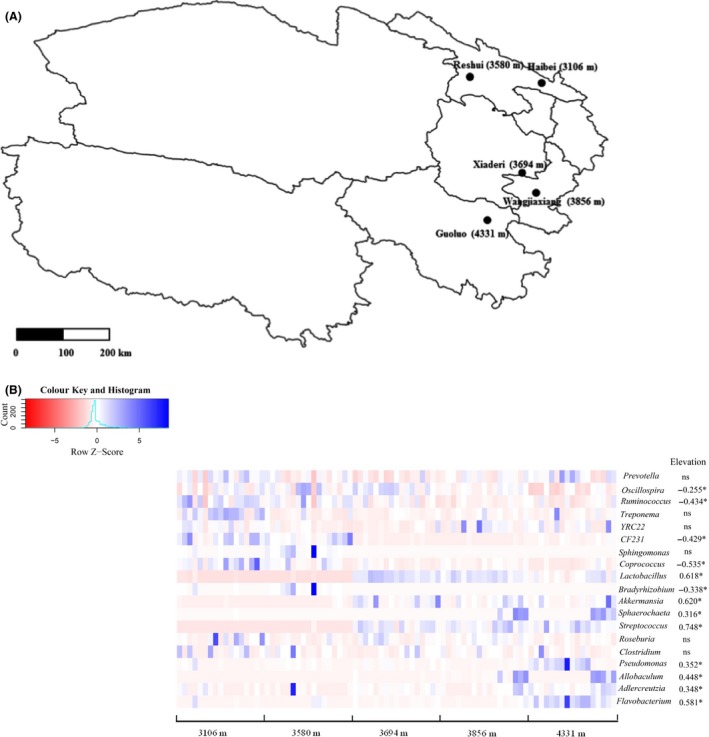
Schematic diagram of five sampling sites (A) and the abundance distribution of major genera in pika gut across elevations and their correlation with elevation (B). The relative abundance of these genera was normalized using *Z*‐score transformation. The blue colour in the heatmap means higher relative abundance of the corresponding genera, while the red colour means less abundant for the relative abundance. Only those genera with mean relative abundance > 0.05% across all samples are shown. Spearman correlation analysis was used to detect the correlation between the relative abundance of these genera and elevation. The symbol (*) means significant correlations with *P *<* *0.05. NS signifies no significance.

In order to understand which bacterial taxa were associated with elevation, Spearman correlation analysis was used to examine the correlations between elevation and bacterial phyla or genera (mean relative abundance > 0.05%). We found that the relative abundances of *Actinobacteria*,* Verrucomicrobia*,* Cyanobacteria* and *Acidobacteria* were positively correlated with elevation, while *Tenericutes* showed a negative association with elevation (Fig. S2). At genus level, elevation was positively associated with eight genera, such as *Lactobacillus*,* Akkermansia*,* Sphaerochaeta*,* Streptococcus*,* Pseudomonas*,* Allobaculum*,* Adlercreutzia* and *Flavobacterium*. However, only five genera (including *Oscillospira*,* Ruminococcus*,* CF231*,* Coprococcus* and *Bradyrhizobium*) showed negative correlations with elevation (Fig. 1).

### Alpha diversity and beta diversity across elevations

**Figure 2:**
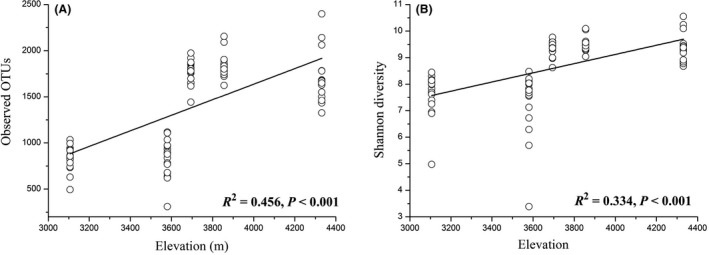
Alpha diversity values (A, observed OTUs; B, Shannon diversity) of the whole pika gut microbiota were significantly correlated with elevation (All *P* values < 0.05).

**Figure 3:**
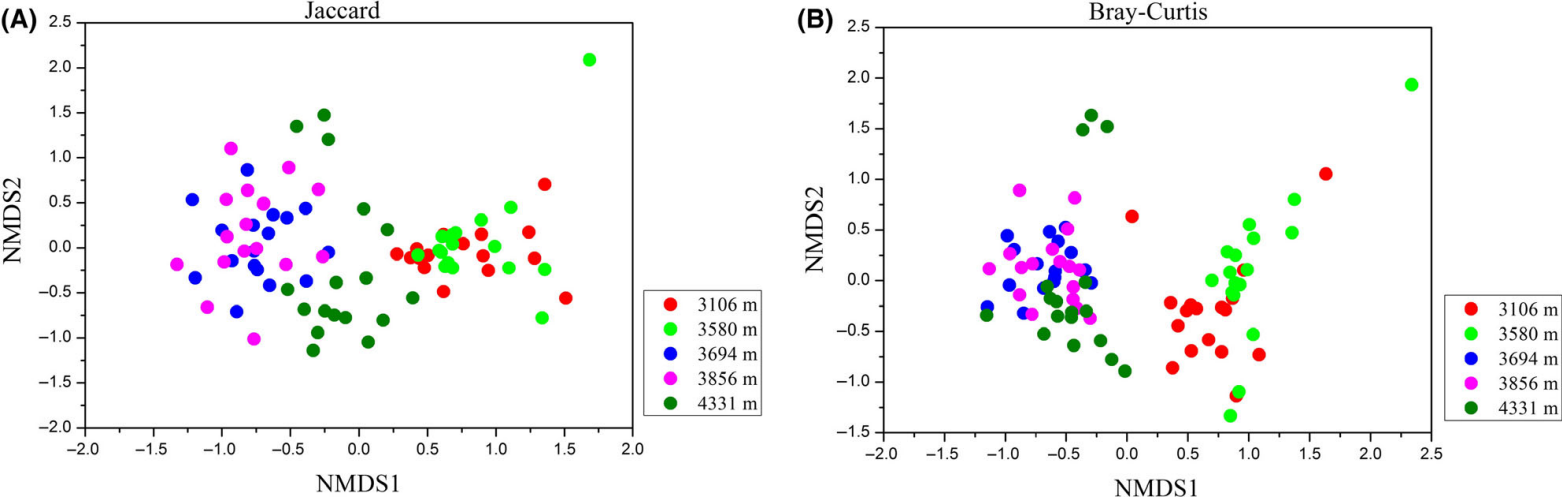
Non‐metric multidimensional scaling (NMDS) plots showing the difference of gut microbiota across elevations at OTU level based on (A) Jaccard and (B) Bray‐Curtis distances.

The alpha diversity (observed OTUs and Shannon diversity) measures of gut microbiota in plateau pikas increased with elevation (Fig. 2), regardless of whether the overall microbial community was considered or just focus on the dominant taxonomic phyla, such as *Firmicutes*,* Bacteroidetes*,* Proteobacteria* and *Spirochaetes* (Fig. S3). However, sex and body weight were not related to alpha diversity (GLM: *P *>* *0.05), indicating high homogeneity in alpha diversity among sex and body weight. Because elevation and air temperature were highly autocorrelative (Fig. S4), we calculated the correlation between temperature and alpha diversity values. We found that temperature was also a good predictor of microbial alpha diversity (Fig. S4). In addition, we found that elevation significantly influenced the gut microbial community composition (Jaccard distance, PERMANOVA *F *=* *23.654, *P *<* *0.001; MRPP Delta* *=* *1.248, *P *<* *0.001) and structure (Bray‐Curtis distance, PERMANOVA *F *=* *14.782, *P *<* *0.001; MRPP Delta* *=* *1.125, *P *<* *0.001) of plateau pikas (Fig. 3). The linear relationship between elevation and PCOA1 of Jaccard or Bray‐Curtis distance matrices (Fig. S5) also confirmed that elevation was a significant factor in shaping the beta diversity of pika gut microbiota. However, sex and body weight also showed no significant impacts on the beta diversity of gut microbiota (all *P *>* *0.05). Mantel test analysis showed that pika gut microbiota was significantly influenced by elevation (Jaccard *r *=* *0.481, *P *<* *0.001; Bray‐Curtis *r *=* *0.549, *P *<* *0.001) and geographical distance (Jaccard *r *=* *0.353, *P *<* *0.001; Bray‐Curtis *r *=* *0.381, *P *<* *0.001). After controlling for the geographical distance between sampling sites, the gut microbiota composition and structure were still correlated with elevation (Jaccard *r *=* *0.369; Bray‐Curtis *r *=* *0.447, *P *<* *0.001).

**Figure 4:**
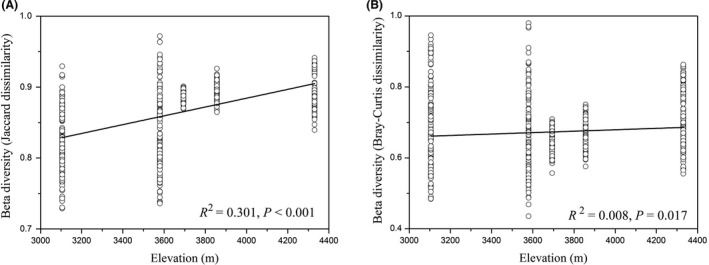
Beta diversity (Jaccard and Bray‐Curtis dissimilarities within each elevation, A–B) values of gut microbiota were significantly correlated with elevation (All *P* values < 0.05).

Similar to alpha diversity, beta diversity (interindividual dissimilarity within each elevational site) values also increased with elevation (both *P *<* *0.05, Fig. 4). In addition, we compared the pairwise differences of pika gut microbiota between altitudes based on Jaccard and Bray‐Curtis distances (Table 1). Community composition and structure had significant differences between elevations except for the site 3106 versus 3580 m based on the Jaccard distances.

**Table 1:** Jaccard and Bray‐Curtis distance dissimilarities showing the differences of pika gut microbiota between elevations

	Jaccard				Bray‐Curtis			
	MRPP		PERMANOVA		MRPP		PERMANOVA	
	Delta	*P*	*F*	*P*	Delta	*P*	*F*	*P*
3106 versus 3580 m	1.208	0.164	1.018	0.316	1.152	**0.003**	3.687	**0.018**
3106 versus 3694 m	1.219	< **0.001**	24.185	< **0.001**	1.11	< **0.001**	28.317	< **0.001**
3106 versus 3856 m	1.225	< **0.001**	22.983	< **0.001**	1.04	< **0.001**	24.195	< **0.001**
3106 versus 4331 m	1.227	< **0.001**	16.683	< **0.001**	1.101	< **0.001**	12.383	< **0.001**
3580 versus 3694 m	1.231	< **0.001**	20.354	< **0.001**	1.016	< **0.001**	45.908	< **0.001**
3580 versus 3856 m	1.236	< **0.001**	19.27	< **0.001**	1.034	< **0.001**	37.38	< **0.001**
3580 versus 4331 m	1.239	< **0.001**	14.898	< **0.001**	1.093	< **0.001**	22.331	< **0.001**
3694 versus 3856 m	1.261	< **0.001**	1.497	**0.007**	0.952	< **0.001**	2.625	**0.01**
3694 versus 4331 m	1.262	< **0.001**	6.629	< **0.001**	1.014	< **0.001**	12.545	< **0.001**
3856 versus 4331 m	1.268	< **0.001**	4.057	< **0.001**	1.034	< **0.001**	6.339	< **0.001**

### Predicted gene functional profiles of pika gut microbiota across elevations

**Figure 5:**
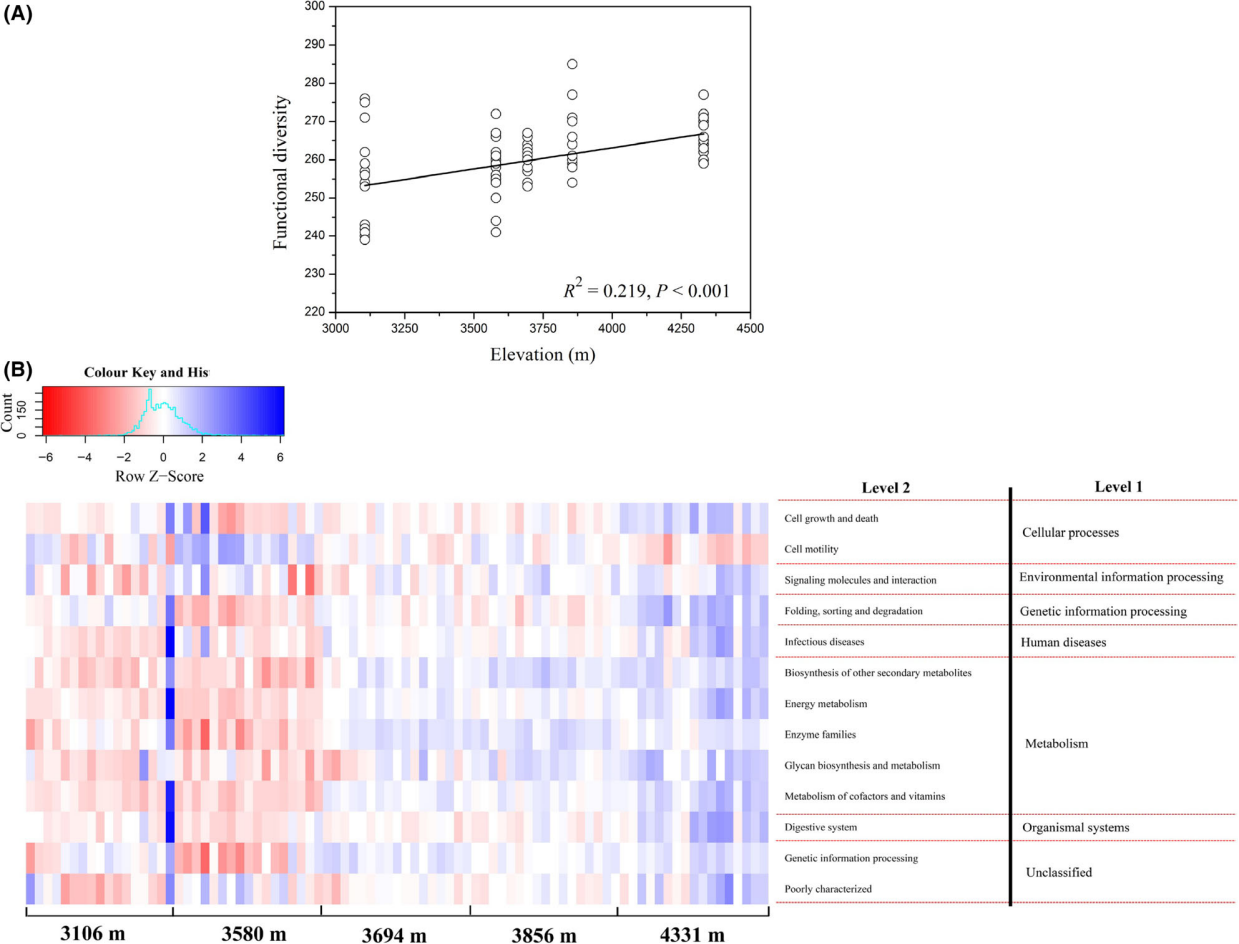
The relationship between elevation and functional diversity or specific gene functions. A. The linear relationship between elevation and functional diversity at level 3. B. The distribution of the predicted gene functions at level 2 that are significantly correlated with elevation. The relative abundance of gene functions was normalized using *Z*‐score transformation. Only those gene functions that correlated with elevation (*r *>* *0.3 or < −0.3, *P *<* *0.01) are shown.

The average nearest sequenced taxon index (NSTI) values for our samples (0.18 ± 0.03) suggested comparable predictive accuracy for our communities as those achieved for those mammalian and fish gut microbiota (Langille *et al*., 2013; Sullam *et al*. 2015). Notably, the functional diversity of gut microbiota at level 3 significantly increased with elevation (Fig. 5A). The NMDS plot based on the Bray‐Curtis dissimilarity matrices of predicted gene functions at level 3 is shown in Fig. S6. We found that the gene functional profiles of pika gut microbiota were significantly impacted by elevation (PERMANOVA, *F* = 9.562, *P *<* *0.001; MRPP, Delta = 0.227, *P *<* *0.001). At level 2, we found that elevation was positively correlated with twelve gene functions, such as energy metabolism, energy families, glycan biosynthesis and metabolism, and cell growth and death, whereas only one gene function (cell motility) showed negative associations with elevation (Fig. 5B). Because the ‘metabolism’ function was the dominant in our predicted metagenomes and also important in food degradation and digestion, we only focused on the difference of the related gene functions across altitudes at level 3. We found a total of 42 gene functions involved in metabolism were correlated with elevation (Fig. S7), while 102 gene functions showed no significant correlations with elevation. In particular, elevation showed positive correlations with 32 gene functions, such as arginine and proline metabolism, nitrogen metabolism, protein kinases and glycosaminoglycan degradation. However, only ten gene functions (i.e. histidine metabolism and primary bile acid biosynthesis) at level 3 were negatively associated with elevation.

### Ecological processes governing the composition of pika gut microbiota

**Figure 6:**
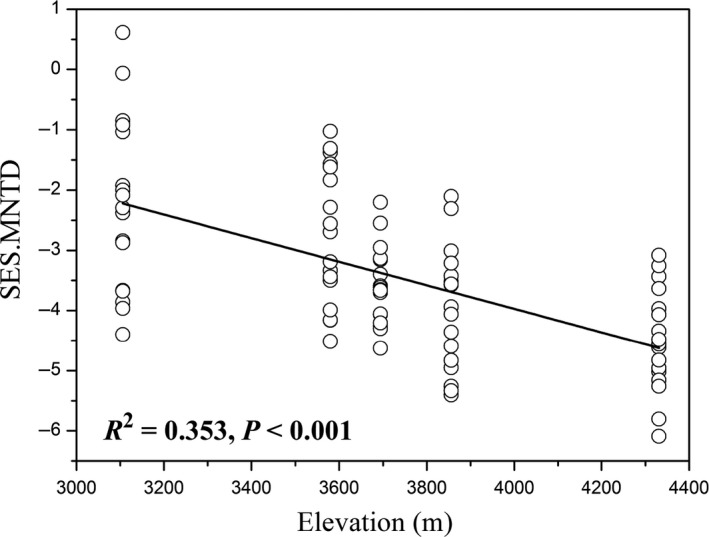
The weighted standardized effect size of the mean nearest taxon distance (SES.MNTD) for the whole pika gut microbiota was significantly correlated with elevation (all *P* values < 0.001).

The Jaccard and Bray‐Curtis distances based on PERMDISP results were significant distinct from the null random expectation within each elevation (Table 2), indicating that pika gut microbiota assembly was primarily governed by deterministic processes based on the taxonomic composition. Phylogenetic signal results (Fig. S1) suggested that it was very suitable to focus on the closely related gut bacterial OTUs in further phylogenetic analysis. Notably, we found that the composition of closely related gut microbiota was also mainly shaped by deterministic processes, as most (84.7%) SES.MNTD values for the local communities were < −2. However, the deterministic processes that determined assembly patterns of pika gut microbiota tended to strengthen significantly (regression models, *P *<* *0.001) with elevation (Fig. 6). This was also found to be true at a particular taxonomic level after separately analysing the three most dominant phyla (*Firmicutes*,* Bacteroidetes* and *Proteobacteria*) (Fig. S8).

**Table 2:** Jaccard and Bray‐Curtis distance‐based significance test of centroid differences between the observed communities and the null model simulations for each elevation

	Actual centroid	Null centroid	*F*	*P*
Jaccard distance				
3106 m	0.563	0.554	1.019	0.003
3580 m	0.561	0.558	0.244	0.006
3694 m	0.577	0.534	695.454	< 0.001
3856 m	0.583	0.533	706.395	< 0.001
4331 m	0.582	0.537	96.028	< 0.001
Bray‐Curtis distance				
3106 m	0.458	0.588	20.322	< 0.001
3580 m	0.448	0.597	39.573	< 0.001
3694 m	0.408	0.544	345.225	< 0.001
3856 m	0.422	0.540	272.183	< 0.001
4331 m	0.460	0.548	26.721	< 0.001

**Figure 7:**
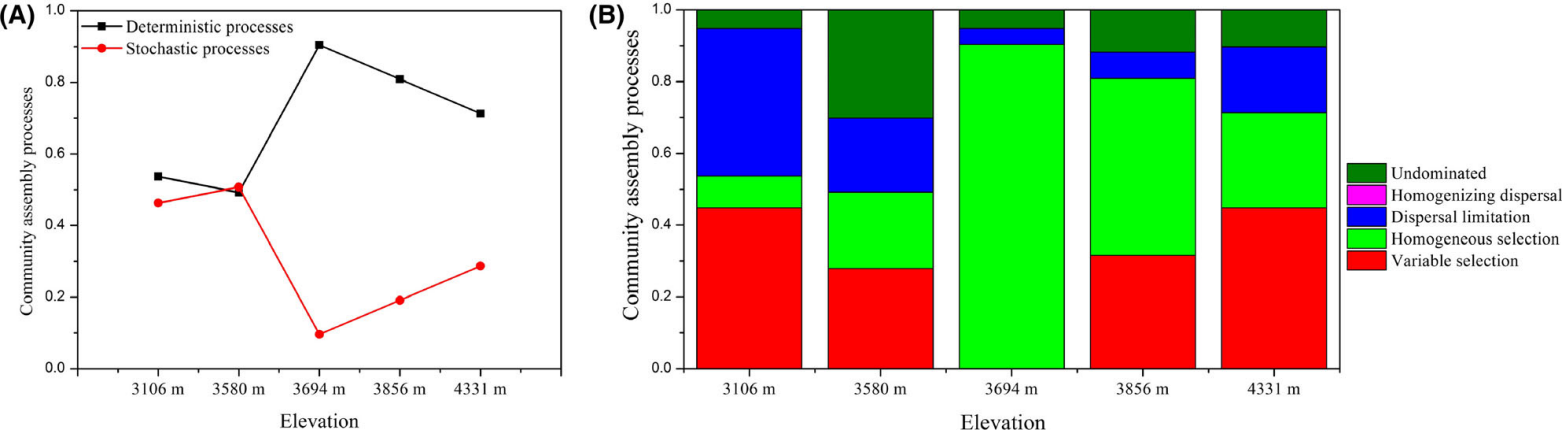
Summary of the contribution of the ecological processes that determine community assembly of pika gut microbiota in different elevations. A. The relative contributions of deterministic processes and stochastic processes in gut microbiota assembly. B. The contributions of variable selection, homogeneous selection, dispersal limitation, homogenizing dispersal and undominated in the assembly of pika gut microbiota.

We quantified the relative contributions of each ecological process in each elevation (Fig. 7). At the low‐altitude regions (3106 and 3580 m), the relative contributions of deterministic processes that govern the assembly of gut microbiota were close to those of stochastic processes. For example, the relative contributions of deterministic processes at the altitude 3106 and 3580 m were 53.7% and 49.3%, respectively, while the corresponding percentages of stochastic processes at these altitudes were 46.3% and 50.7%. In contrast, deterministic processes play dominant roles in the assembly of pika gut microbiota at the high‐altitude regions (3694 m, 90.4%; 3856 m, 80.9%; 4331 m, 71.3%) and were higher than those from the low‐altitude regions. Variable selection and homogeneous selection belong to deterministic processes, while dispersal limitation, homogenizing dispersal and undominated belong to stochastic processes (Dini‐Andreote *et al*., 2015; Tripathi *et al*., 2018). Thus, we further analysed the difference of finer processes between elevations. We found that the fraction of variable selection and dispersal limitation decreased from the altitude 3016 m to the 3694 m, whereas these ecological processes increased gradually from 3694 to 4331 m. In contrast, the fraction of homogeneous selection increased from the altitude 3016 m to the 3694 m, whereas the ecological processes decreased gradually from 3694 to 4331 m.

### Nucleotide sequence accession numbers

The original 16S rRNA gene data were available at the European Nucleotide Archive by accession number PRJEB26673 ( http://www.ebi.ac.uk/ena/data/view/PRJEB26673).

## Discussion

Investigating community assembly mechanisms is a hot topic in the field of community ecology. However, most previous reports focused on the plant and animal communities, relatively few studies have explored the assembly of microbial communities, especially regarding host–gut microbiota. Although several studies have revealed that assembly processes of gut microbiota in fish (Yan *et al*., 2016) and fruit flies (Adair *et al*., 2018), the results on the dominant ecological processes responsible for gut microbiota assembly were contradictory. Besides, previous studies mainly focused on the gut community assembly of fish and arthropods, and the knowledge on the mammalian gut microbiota assembly is rather lacking. Although there are several reports on the gut microbiota composition and diversity in humans and lizards across short elevational gradients (Li and Zhao, 2015; Zhang *et al*., 2018), we are the first to study the microbiota assembly mechanisms in the mammalian gut along a longer altitude span. Our results showed that elevation is a significant factor in shaping pika gut microbiota diversity, even after controlling for host sex, body weight and geographical distances. In addition, we found that deterministic processes dominated the assembly of gut microbial communities in the high‐altitude (more than 3694 m) pikas, while the percentages of stochastic and deterministic processes were very close in the low‐altitude (3106 and 3580 m) pikas. These results greatly expand our understanding on gut microbiota assembly patterns in mammals.

### High‐altitude pikas possess more microbes that consist of potential probiotics, pathogens and commensal bacteria

The relative abundances of thirteen bacterial genera in the pika gut showed significant correlations with elevation (Fig. 1). Among these bacteria, eight genera (i.e. *Lactobacillus*,* Akkermansia*,* Streptococcus* and *Pseudomonas*) increased with elevation, indicating that microbes from these genera in the pika gut may well adapt to the harsh, cold environment at higher elevations. Those microbes that were enriched in the high‐altitude pikas probably provide important functions for hosts. For example, several members of *Lactobacillus* may form biofilms in the gut microbiota, thus increasing resistance to temperature, pH and mechanical forces. These features allow them to persist in extreme environments and maintain ample populations (Salas‐Jara *et al*., 2016). In addition, some members of *Lactobacillus* (i.e. *Lactobacillus rhamnosus* GG) can prevent pathogen invasions and help treat diarrhoea in the human guts (Saxelin, 2008; Martín *et al*., 2013; Inglin, 2017). Another potential beneficial genus is *Akkermansia*. One specific species (*Akkermansia muciniphila*) from this genus can adhere to the gut mucosal interface between the lumen and host cells, and it is a highly specialized mucin‐degradation microbe (Belzer and de Vos, 2012). Evidence showed that these mucus‐colonizing bacteria may protect hosts again intestinal pathogens invasion and is beneficial for the microbiota restoration (Reid *et al*., 2011). Several studies have revealed that *Akkermansia muciniphila* coincides with improved metabolic ability, and showed negative correlations with metabolic disorder or diseases (Everard *et al*., 2013; Derrien *et al*., 2017). Taken together, we found that some potential probiotics were enriched in the high‐altitude pikas. These genera in the pika gut are likely to help pikas improve metabolic ability and maintain host health in order to adapt to the harsh, cold and hypoxic high‐altitude environments. Further research is needed to accurately evaluate the ecological functions of these bacteria in pika guts.

In contrast to the observed potential probiotics, we found that several potential pathogenic bacterial genera (i.e. *Pseudomonas and Flavobacterium*) were also positively correlated with elevation. Some members of the genus *Pseudomonas* demonstrate a high metabolic diversity and consequently can colonize a wide range of niches (Madigan and Martinko, 2005). Some species from *Pseudomonas* have been detected in humans (i.e. *P. aeruginosa*) (Oliver *et al*., 2000), animals (i.e. *Pseudomonas* sp.KUMS3) (Kumaran *et al*., 2009) and plants (*P. tolaasii*) (Brodey *et al*., 1991) and are able to cause various diseases for these organisms. Several species of *Flavobacterium* lead to various diseases in fish. For example, *F. psychrophilum* is the causative agent of the bacterial cold water disease on salmonid fish (Nematollahi *et al*., 2003). In addition, both *F. branchiophilum* and *F. succinicans* may cause bacterial gill disease in rainbow trout (*Oncorhynchus mykiss*) (Good *et al*., 2015). Although these pathogens had the potential disease risk, their functional roles in the pikas remain still unknown.

In addition to the potential probiotics and pathogens, we also found that some commensal bacteria enriched in the pika gut. For example, species from *Streptococcus* are generally common bacterial taxa in human skin, mouth, throat and intestine (van der Mee‐Marquet *et al*., 2008; Zaura *et al*., 2009; Chaffanel *et al*., 2018). Our previous study also showed that *Streptococcus* was one of the dominant bacterial taxa in pika mouths (Li *et al*., 2017a), indicating that this genus probably widely distributes in mammalian microbiota. Since some species in this genus have been reported to be involved in pyruvate metabolism in humans (Jorth *et al*., 2014), we speculate they play important roles in the gut niche of high‐altitude pikas.

### The alpha, beta and functional diversity of gut microbiota increase with elevation

In general, ecologists assume that ecosystems containing more species diversity would be more stable and may exhibit higher levels of ecosystem performance and functions (Elton, 1958; Margalef, 1963; McNaughton, 1977). Although species in diverse ecosystems can interact differently, evidence showed that high species diversity provides more functional redundancy and buffer ecosystem functions against species loss or extinctions (Bannar‐Martin *et al*., 2018; Louca *et al*., 2018). In host–microbe systems, a diverse bacterial community may contribute a distinct set of digestive enzymes (Werner *et al*., 2011) and improve food processing and digestion. High microbial diversity is generally associated with strong metabolic ability and stability. For instance, gut microbiota diversity in humans increased fermentation efficiency of dietary fibre and promotes the stability of gut ecosystems (Tap *et al*., 2015). Besides, increased microbial diversity in human gut also reflects a better state of health and stronger metabolic capacity (Clarke *et al*., 2014). In our study, species diversity (or alpha diversity) of pika gut microbiota increased with elevation, and the results were not consistent with the findings in Li and Zhao (2015) and Zhang *et al*. (2018), who demonstrated that there were no significant differences in alpha diversity measures of human and lizard gut microbiotas at different altitudes. We speculate that the gut communities of high‐altitude pikas probably possess higher degradation ability for indigestible plant polysaccharides. Two reasons may explain this inference. First, we have demonstrated that alpha diversity of pika gut microbiota was positively associated with cellulolytic activity (Li *et al*., 2018). Second, high‐altitude plateau pika harbours higher gut microbial diversity and stronger fermentation capacity than low‐altitude Daurian pika (*Ochotona daurica*) (Li *et al*., 2018). However, most reports showed that mammalian gut is considered sterile at birth, thus pikas from high‐altitude regions select for more diverse bacterial colonists from surrounding environment (i.e. plant and soil) in order to improve gut function. Our results suggest that the predicted gene functional profiles of pika gut microbiota were significantly impacted by elevation. In particular, those gene functions involved in energy metabolism, energy families, and glycan biosynthesis and metabolism were enriched in the high‐altitude pikas, indicating the high‐altitude pikas are likely to improve metabolic functions of gut microbiota in order to cope with cold and hypoxic plateau environment. However, our results were only based on the predicted metagenome, which may not be representative of the actual gut bacterial functions. Further research is needed to directly sequence the pika gut metagenome for exploring the roles of these gene functions in animal environmental adaptability.

Elevation is a complex environmental gradient where many environmental factors change, and we found that the beta diversity of gut microbial communities was significantly different across altitudes, similar to other studies (Li and Zhao, 2015; Zhang *et al*., 2018). Multiple factors (e.g. oxygen, air temperature, atmospheric pressure and diet resources) associated with elevation may influence the gut microbiota structure. Although pika is homothermal animal, evidence shows that external cold temperature may still influence the gut microbiota composition in homothermal rodents (Stevenson *et al*., 2014). Temperature may indirectly impact microbiota structure by mediating microbe–microbe interactions. For example, priority effects and temperature change the abundance of microbial community members, so that if certain bacteria colonize first, they may restrain the abundance of other microbes, but this process is dependent on temperature (Tucker and Fukami, 2014). In addition, hyperbaric pressure, which is a typical feature of high altitude, has been demonstrated as an important factor that strongly mediates the composition of gut microbiota in mammals (Adak *et al*., 2013; Zhang *et al*., 2018). High‐altitude pikas harbour less diverse diet resource compared with low‐altitude ones (Li *et al*., 2018), and thus, diet availability and composition may influence the gut microbiota composition. Besides, the differences of gut microbiota structure between altitudes are also attributed to host genetics, geographical distance and some other unknown factors. It is strange that the gut microbiotas of high‐elevation pikas from 3694 to 4331 m had a clear structural separation with those in the low‐elevation pikas from 3106 to 3580 m (Fig. 3). We speculate that the possible reason is that a certain elevational range between 3580 and 3694 m is a demarcation line for obvious changes in pika physiology, which may enormously influence the structures of gut microbiota. Further research should demonstrate this hypothesis. Notably, elevation significantly explained more variations than geographical distance in shaping the gut microbial communities, indicating that vertical environmental filtering should be more pronounced than horizontal dispersal. However, because air temperature and elevation are autocorrelative, it is impossible to uncover the relative contributions of air temperature and elevation on pika gut microbiota. Further research should disentangle the effect of each factor associated with elevation on mammalian gut microbiota based on control experiments.

Beta diversity of gut microbiota within each elevation also increased with elevation, indicating that interindividual gut microbial communities are more dissimilar on the high‐altitude regions. We enumerate three hypotheses for further testing in future. First, although diet resources on the high‐altitude regions are more homogeneous and are less diverse compared with those on the low‐altitude areas, each pika individual still can possess their own food preferences and choose personalized diet species. Second, high‐altitude environmental pressure may cause different physiological and immune responses for each host individual (Adak *et al*., 2013), thus probably indirectly increasing the dissimilarity of individual gut microbiota. Finally, environmental (i.e. plant and soil) microbes at different altitudes may also influence the similarity of pika gut microbiota.

### Environmental filtering increases with elevation for gut microbiota assembly

The null model test (PERMDISP) results showed that gut microbiota assemblages of each elevation differ significantly from the null random expectation based on Jaccard or Bray‐Curtis dissimilarity matrices (Table 2). These data suggest that the composition of pika gut microbiota primarily assemble deterministically rather than stochastically. Our results are congruent with those in several fish microbiota reports (Schmidt *et al*., 2015; Yan *et al*., 2016), which also showed gut microbiota assembly was mainly governed by deterministic processes. However, our previous studies have found that substantial individual variations in the gut microbiota of pikas (Li *et al*., 2016b, 2016c, 2016d), indicating that stochastic processes also contribute to the gut community assembly. Thus, we further used a new ecological framework to quantify the relative importance of each ecological process in governing the gut community assembly across elevations.

Ecological process inference requires a significant phylogenetic signal (Stegen *et al*., 2012). We detected significant phylogenetic signal across relatively short phylogenetic distance (Fig. S1). This finding indicates more closely related bacterial/OTU taxa have more similar niche preferences related to elevation. Although some imperfection of methods (e.g. DNA extraction methods, PCR bias and sequencing errors) may influence the estimation of ecological processes using this framework (Stegen *et al*., 2015), this method is considered effective and widely applied in analysing the gut microbiota assembly of human, fish, fruit fly (Martinez *et al*., 2015; Yan *et al*., 2016; Adair *et al*., 2018) and other environmental microbiota (Wang *et al*., 2012). Based on the phylogenetic composition, we found that environmental filtering (or phylogenetic clustering) dominates the microbiota assemblages in the pika gut ecosystems at local scales, as indicated by negative SES.MNTD (Wang *et al*., 2013). However, the relative importance of environmental filtering tends to strengthen along elevations, indicating that elevation acts as a strict environmental filter and cause phylogenetic clustering. Environmental filtering is a pivotal determinant of community assembly (Webb *et al*., 2002), and this has led to phylogenetic clustering of closely related OTUs in pika gut. Intestinal tract mass of plateau animals is always directly proportional to elevation (Han *et al*., 2016), thus providing additional space for niche filling, and finally leading to an increase in deterministic processes. In addition, high‐altitude extreme environments can improve gut‐associated immune system (Adak *et al*., 2013) and finally resulting in similar gut environment as a result of deterministic processes. Our results also found that not all pika individual gut microbiotas were governed by deterministic assembly, indicating that pika gut microbiota is dynamic and may be influenced by some other unknown factors.

In addition, we found that high‐altitude pikas (3694, 3856 and 4331 m) were dominated by homogeneous selection, confirming that similar host or environmental factors lead to similar gut microbiota. By contrast, those low‐altitude pikas (3106 and 3580 m) had more fraction of dispersal limitation. This suggests that they harbour higher frequency of microbes transmission, probably through physical contact, kiss, caecotrophy behaviour (Yatsunenko *et al*., 2012; VanderWaal *et al*., 2014; Li *et al*., 2016b) or other indirect transmission methods (e.g. environmental transmission) (Nunn *et al*., 2011). More limited transmission for gut microbes may be one important reason of higher beta diversity within high‐altitude pikas.

In conclusions, we are the first to study gut microbiota assembly in high‐altitude mammals along environmental gradients on ‘the Roof of the World’—Qinghai‐Tibet Plateau. Our results showed that the alpha, beta and functional diversity of gut microbiota significantly increase with elevation, indicating elevation is a pivotal determinant of community assembly. Deterministic processes dominated the assembly of gut microbial communities in the high‐altitude (more than 3694 m) pikas, while the percentages of stochastic and deterministic processes were very close in the low‐altitude (3106 and 3580 m) pikas. In addition, the relative contributions of environmental filtering responsible for local gut communities increased with elevation. These results greatly expand our understanding for gut microbiota assembly patterns in wild animals from extreme plateau environments. Due to the deterministic assembly, the gut microbiome of wild pikas can be manipulated to improve animal health for wildlife reserves.

## Experimental procedures

### Sample collection

We collected caecal content samples of plateau pikas from five different sampling sites on the Qinghai‐Tibet Plateau in the summer of 2014–2016. These five sites were Guoluo (4331 m ASL), Wangjiaxiang (3856 m ASL), Xiaderi (3694 m ASL), Reshui (3580 m ASL) and Haibei (3106 m ASL). These habitat types are typical alpine meadows. The plant communities of these habitats were all dominated by *Kobresia humilis*, followed by *Ajuga lupulina*,* Leontopodium nanum*,* Potentilla anserina*,* Poa annua* L., *Pedicularis* sp., *Oxytropis* sp., *Tibet Lancea* and *Elymus nutans Griseb*. The potential food resources (or plant community compositions) are very similar for pikas across elevations based on our survey (Fig. S9). We randomly captured 17 pika individuals from each sampling site. The methods of sample collection and preservation in different sampling sites were identical. Briefly, upon capture, each pika was euthanized and then dissected. Thereafter, caecal contents were immediately collected into 50‐ml sterile tubes and frozen at −20°C in a portable freezer. Finally, all samples were transferred to our laboratory within 24 h and stored at −40°C until DNA extraction. The air temperature of sampling sites was recorded during sampling. The detailed information of each sample is listed in Table S1.

All animal experiments were approved by the Animal Care and Ethics Committee of Northwest Institute of Plateau Biology, Chinese Academy of Sciences (NWIPB‐2014‐123). The experimental procedures followed the relevant guidelines strictly.

### DNA extraction, PCR amplification and high‐throughput sequencing

The Ezup DNA extraction kit for soil (Sangon Biotech, China) was used to extract total DNA from the caecal contents following the manufacturer's instructions. A negative control (sterile water) was used in our extraction process. DNA concentration and quality were checked using a Nanodrop 2000 Spectrophotometer (Thermo Scientific, Waltham, IL, USA). The procedures of PCR amplification, gel extraction and sequencing were described previously (Li *et al*., 2016c). Briefly, universal primers 515F (5′‐GTGYCAGCMGCCGCGGTA‐3′) and 909R (5′‐CCCCGYCAATTCMTTTRAGT‐3′) with 12 nt unique barcode at 5′‐end of 515F were used to amplify the V4 and V5 region of microbial 16S rRNA gene (Tamaki *et al*., 2011). Each PCR contained a negative control where sterile water without any DNA was used. PCR amplification was performed in twice for each DNA sample in order to minimize PCR bias. The detailed procedures of PCR amplification and gel extraction were described previously (Li *et al*., 2016b). Finally, the resulting amplicons were mixed with equal molar and then sequenced using 250‐bp paired‐end Illumina MiSeq systems (Illumina, San Diego, CA, USA).

### Bioinformatics analysis

The original sequences were processed using QIIME Pipeline‐Version 1.9.0 ( http://qiime.org/scripts/index.html) (Caporaso *et al*., 2010). Briefly, sequences were assigned to each sample only if they perfectly matched their unique barcodes. Barcodes were not allowed to contain the wrong base. Thereafter, the paired‐end reads were joined with flash‐1.2.8 software (Magoc and Salzberg, 2011) only if they had at least 20 base overlaps. Sequence filtering and analysis followed the methods of Li *et al*. (2016b). Briefly, sequences with barcode ambiguities, with read length < 300 bp and with average quality score < 30 were excluded. Chloroplast sequences were detected and removed using metaxa2 software tool (Bengtsson‐Palme *et al*., 2015). Thereafter, the aligned 16S rRNA gene sequences were subjected to a chimera check using the Uchime algorithm (Edgar *et al*., 2011). The remaining sequences were clustering into operational taxonomic units (OTUs) at 97% sequence similarity with an open‐reference OTU picking method using the Uclust algorithm (Edgar, 2010). Singletons were also removed. Negative controls were not detected any PCR bands, indicating minimum contamination in our study. The most abundant sequence for each OTU was selected as the OTU's representative sequence, and these sequences were aligned against the Greengenes 13_8 reference database (DeSantis *et al*., 2006) using PyNAST(Caporaso *et al*., 2009). Thereafter, taxonomic classification was performed using the Ribosomal Database Project (RDP) classifier with a standard threshold of 80% (Wang *et al*., 2007). Taxonomic profiles were evaluated at the phylum and genus level.

Those sequences not classifying to bacteria were filtered. Thereafter, each sample was rarefied to 3926 sequences in order to minimize the influence of sequencing depth on bacterial community diversity. In order to assess alpha diversity, observed OTUs and Shannon diversity were calculated. To evaluate beta diversity indices, Jaccard (for community composition) (Jaccard, 1912) and Bray‐Curtis distances (for community structure) (Bray and Curtis, 1957) were calculated using QIIME pipeline. Jaccard distance was based on the presence/absence of OTUs, whereas Bray‐Curtis distance was based on the relative abundance of OTUs. Beta diversity (interindividual dissimilarities within each elevational site) was examined using the two dissimilarity metrics. Principal coordinates analysis (PCoA) values of the dissimilarity metrics were also calculated. Differences in overall bacterial community structure across altitudes were visualized using the non‐metric multidimensional scaling (NMDS) plots of the two distance matrices.

Functional metagenomes were predicted based on the 16S rRNA gene sequences using PICRUSt (Langille *et al*., 2013). To evaluate the accuracy of our metagenomic prediction, we calculated the Nearest Sequenced Taxon Index, which was used to indicate the average divergence between each 16S rRNA sequence within OTU and their closest reference sequenced genome. The predicted gene functions were evaluated at levels 2 and 3. The observed number of gene functions (functional diversity) at level 3 was calculated. Bray‐Curtis distance matrices were calculated based on the gene‐abundance table at level 3. Differences in overall gene functional profiles across altitudes were visualized using the non‐metric multidimensional scaling (NMDS) plots of the Bray‐Curtis distance matrices. Notably, at level 3, we only focused on the difference of gene functions associated with metabolism across altitudes, because these gene functions were dominant in predicted metagenomes and were important in food degradation and digestion.

### Null model and phylogenetic analysis

Mean nearest taxon distance (MNTD) and SES.MNTD were used to qualitatively evaluate the deterministic processes or stochastic processes of community composition (Meyerhof *et al*., 2016) based on phylogenetic analysis using the ‘picante’ package in r. The phylogenetic signals (Mantel correlation, Pearson's *r*,* P *<* *0.001) extended across relatively short phylogenetic distance (Fig. S1), and thus, it was completely appropriate to quantify phylogenetic relationship among the closest bacterial relatives (Stegen *et al*., 2012) in current study. SES.MNTD is the differences between the observed MNTD and mean expected MNTD divided by the standard deviation of random expected values. Because microbial communities often consist of few high‐abundance OTUs and a majority of low‐abundance OTUs, we only focused on the weighted‐abundance SES.MNTD values in the following analysis. If SES.MNTD values are between −2 and 2, then community composition is mainly governed by stochastic processes. If SES.MNTD values are > 2 or < −2, then deterministic processes (phylogenetic overdispersion or phylogenetic clustering) play a dominant role in structuring the microbial community (Meyerhof *et al*., 2016). Moreover, a larger absolute magnitude of SES.MNTD value (|SES.MNTD|) signifies greater contributions of deterministic processes (Stegen *et al*., 2012, 2013).

To test which ecological processes (variable selection, homogeneous selection, dispersal limitation, homogenizing dispersal or undominated) may govern bacterial community composition between elevations, we followed the methods of Stegen *et al*. (2013) in order to calculate the potential ecological processes. Briefly, we calculated the phylogenetic diversity of bacterial communities between a pair of samples. R package was applied to quantify the weighted beta nearest taxon index (β‐NTI). The combination of β‐NTI and Bray‐Curtis‐based Raup‐Crick (RC_bray_) was further used to infer the relative importance of major ecological processes governing the bacterial communities. Values of β‐NTI > 2 or < −2 represent community turnover determined by the variable selection or homogeneous selection. If −2 < β‐NTI < 2 and RC_bray_ > 0.95 or < −0.95, then the community turnover is determined by dispersal limitation or homogenizing dispersal respectively. If 2 < β‐NTI < 2 and −0.95 < RC_bray_ < 0.95, indicating that community turnover is not the result of a single dominant process (referred to as ‘undominated’ or ‘drift’ processes) (Stegen *et al*., 2015). Homogeneous selection means that a coincident selective environment among local scales leads to similar community composition. The variable selection represents differences in selective environment among local scales cause differences in community composition. The ecological significance of homogenizing dispersal is that the broad dispersal rate leads to similar community composition among local scales. Dispersal limitation signifies that limited dispersal rate causes divergence in community composition. Ecological drift is derived from stochastic changes (i.e. birth or death) in population sizes (Vellend, 2010; Stegen *et al*., 2013). Notably, variable selection and homogeneous selection belong to deterministic processes, while dispersal limitation, homogenizing dispersal and undominated belong to stochastic processes (Dini‐Andreote *et al*., 2015; Tripathi *et al*., 2018).

### Statistical analysis

Statistical analysis included the following several aspects: (i) multiple‐response permutation procedure (MRPP) and permutational multivariate analysis of variance (PERMANOVA) (Yan *et al*., 2016) were used to elucidate whether the community structures were significantly different across elevations based on the Jaccard and Bray‐Curtis distance matrices. The model also included the variables host sex and body weight. MRPP and PERMANOVA were also used to evaluate the differences of the predicted gene functional profile at level 3 based the Bray‐Curtis distance matrices; (ii) Spearman correlation analysis was used to examine the strength and significance of relationships between altitude and major phyla, genera. Only those phyla or genera with mean relative abundance > 0.05% across all samples were considered. In addition, the relationship between those predicted gene functions (at level 3) and elevation was tested using Spearman correlation analysis, and we only focused on those gene functions associated with metabolism; (iii) generalized linear models were used to explore the relationship between elevation and alpha diversity, beta diversity, functional diversity or SES.MNTD; (iv) elevation and geographical distance were transformed as Euclidean distance matrices, and then, mantel test and partial Mantel test was used to examine the correlation between distance matrices (Jaccard and Bray‐Curtis) of gut microbiota and elevation, geographical distance using Spearman correlation analysis; (v) a null model analysis was performed to investigate whether the observed beta diversity values (Jaccard and Bray‐Curtis distance matrices) are distinct from the null expectation based on permutational analysis of multivariate dispersion (PERMDISP) (Chase *et al*., 2011). If the observed Jaccard or Bray‐Curtis distance is significantly different from the null model, then deterministic processes dominate the microbial community assembly. In contrast, if the observed distance metrics are not significantly distinguishable from the random null model, stochastic processes play a major role in the community composition. All statistical analyses were performed using the ‘vegan’, ‘stats’, ‘phyloseq’ and ‘picante’ in r programs (R Foundation for Statistical Computing, Vienna, Austria).

## Conflict of interest

None declared.

## Author contributions

HL designed the research; HL and JQ contributed to sample collection; HL performed the molecular work, finished the data analysis and wrote the original manuscript. All the authors contributed to the revision of the manuscript.

## Supporting information


**Fig. S1** Mantel correlation between the pairwise matrix of OTU niche distances and the phylogenetic distances in pika gut microbiota with 999 permutations. Significant correlations (*P *<* *0.05) of phylogenetic signals in species ecological niches are marked as solid circles, whereas non‐significant correlations are labeled as hollow circles.
**Fig. S2** The abundance distribution of major phyla in pika gut across elevations and their correlation with elevation. The relative abundance of these phyla was normalized using Z‐score transformation. Only those phyla with mean relative abundance > 0.05% across all samples are shown.
**Fig. S3** Alpha diversity (observed OTUs and Shannon diversity) values of the dominant phyla Firmicutes, Bacteroidetes, Proteobacteria and Spirochaetes were significantly correlated with elevation (All *P* values < 0.05).
**Fig. S4** The linear regression relationship between temperature and elevation, observed OTUs or Shannon diversity.
**Fig. S5.** Relationship between elevation and principal coordinate axis 1(PCoA1) from Jaccard or Bray‐Curtis dissimilarities for pika gut microbiota. PCoA1 is significantly correlated with Jaccard or Bray‐Curtis distance.
**Fig. S6** Non‐metric multidimensional scaling (NMDS) plots showing the difference of gene functional profiles across elevations at level 3 based on Bray‐Curtis distance.
**Fig. S7** The abundance distribution of the predicted gene functions associated with metabolism at level 2 across elevations. The relative abundance of gene functions was normalized using Z‐score transformation. Only those gene functions that correlated with elevation (*r* > 0.3 or < −0.3, *P *<* *0.01) are shown (all *P* values < 0.001).
**Fig. S8** The weighted standardized effect size of the mean nearest taxon distance (SES.MNTD) for the three dominant phyla Firmicutes, Bacteroidetes, Proteobacteria was significantly correlated with elevation (all *P* values < 0.001).
**Fig. S9** The composition of plant communities in each elevation. Only nine most abundant plant species were shown.
**Table S1**. The detailed sample information.
