# Supplementary material for: Environmental filtering increases with elevation for the assembly of gut microbiota in wild pikas
Source: Microb Biotechnol. 2019 Jun 10;12(5):976–92. doi: 10.1111/1751-7915.13450 (PMC6680628; doi:10.1111/1751-7915.13450)
Supplement: Supplementary file 1 — Fig. S1 Mantel correlation between the pairwise matrix of OTU niche distances and the phylogenetic distances in pika gut microbiota with 999 permutations. Significant correlations (P < 0.05) of phylogenetic signals in species ecological niches are marked as solid circles, whereas non‐significant correlations are labeled as hollow circles. Fig. S2 The abundance distribution of major phyla in pika gut across elevations and their correlation with elevation. The relative abundance of these phyla was normalized using Z‐score transformation. Only those phyla with mean relative abundance > 0.05% across all samples are shown. Fig. S3 Alpha diversity (observed OTUs and Shannon diversity) values of the dominant phyla Firmicutes, Bacteroidetes, Proteobacteria and Spirochaetes were significantly correlated with elevation (All P values < 0.05). Fig. S4 The linear regression relationship between temperature and elevation, observed OTUs or Shannon diversity. Fig. S5. Relationship between elevation and principal coordinate axis 1(PCoA1) from Jaccard or Bray‐Curtis dissimilarities for pika gut microbiota. PCoA1 is significantly correlated with Jaccard or Bray‐Curtis distance. Fig. S6 Non‐metric multidimensional scaling (NMDS) plots showing the difference of gene functional profiles across elevations at level 3 based on Bray‐Curtis distance. Fig. S7 The abundance distribution of the predicted gene functions associated with metabolism at level 2 across elevations. The relative abundance of gene functions was normalized using Z‐score transformation. Only those gene functions that correlated with elevation (r > 0.3 or < −0.3, P < 0.01) are shown (all P values < 0.001). Fig. S8 The weighted standardized effect size of the mean nearest taxon distance (SES.MNTD) for the three dominant phyla Firmicutes, Bacteroidetes, Proteobacteria was significantly correlated with elevation (all P values < 0.001). Fig. S9 The composition of plant communities in each elevation. Only nine most abundant plan [file MBT2-12-976-s001.pdf]

Supporting Information

Environmental filtering increases with elevation for the assembly of gut microbiota in wild pikas

Huan Li[1,4*], Rui Zhou[1], Jianxiao Zhu[2], Xiaodan Huang[1], Jiapeng Qu[3, 4*]

1. School of Public Health, Lanzhou University, Lanzhou 730000, China
2. State Key Laboratory of Grassland Agro-ecosystems，College of Pastoral Agriculture Science and Technology, Lanzhou University, Lanzhou, 730020, P. R. China
3. Key Laboratory of Adaptation and Evolution of Plateau Biota, Northwest Institute of Plateau Biology, Chinese Academy of Sciences, Xining, Qinghai, 810008, China
4. Key laboratory of restoration ecology of cold area in Qinghai Province, Xining, Qinghai 810008, China

[*]Corresponding authors, H.L., lihuanzky@163.com; J. Qu, jpqu@nwipb.cas.cn

Running title: Gut microbiota assembly of pika across elevations

Table S1. The detailed sample information

Sample ID	Sex	Body weight	Elevation(m)	Sampling site	Temperature (°C）	Geographic coordinates
EP1	Female	215	3106	Haibei	22.4	101.3575°E, 37.7567° N
EP2	Male	135	3106	Haibei	22.4	101.3575°E, 37.7567° N
EP3	Male	152	3106	Haibei	22.4	101.3575°E, 37.7567° N
EP4	Female	148	3106	Haibei	22.4	101.3575°E, 37.7567° N
EP5	Male	135	3106	Haibei	22.4	101.3575°E, 37.7567° N
EP6	Female	102	3106	Haibei	22.4	101.3575°E, 37.7567° N
EP7	Female	108	3106	Haibei	22.4	101.3575°E, 37.7567° N
EP8	Male	156	3106	Haibei	22.4	101.3575°E, 37.7567° N
EP9	Female	168	3106	Haibei	22.4	101.3575°E, 37.7567° N
EP10	Female	59	3106	Haibei	22.4	101.3575°E, 37.7567° N
EP11	Female	159	3106	Haibei	22.4	101.3575°E, 37.7567° N
EP12	Female	150	3106	Haibei	22.4	101.3575°E, 37.7567° N
EP13	Male	149	3106	Haibei	22.4	101.3575°E, 37.7567° N
EP14	Female	50	3106	Haibei	22.4	101.3575°E, 37.7567° N
EP15	Male	50	3106	Haibei	22.4	101.3575°E, 37.7567° N
EP16	Male	154	3106	Haibei	22.4	101.3575°E, 37.7567° N
EP17	Male	142	3106	Haibei	22.4	101.3575°E, 37.7567° N
EP18	Male	172	3580	Reshui	17.8	100.4719 °E, 37.5086° N
EP19	Female	215	3580	Reshui	17.8	100.4719 °E, 37.5086° N
EP20	Female	178	3580	Reshui	17.8	100.4719 °E, 37.5086° N
EP21	Female	180	3580	Reshui	17.8	100.4719 °E, 37.5086° N
EP22	Male	172	3580	Reshui	17.8	100.4719 °E, 37.5086° N
EP23	Male	168	3580	Reshui	17.8	100.4719 °E, 37.5086° N
EP24	Female	221	3580	Reshui	17.8	100.4719 °E, 37.5086° N
EP25	Male	154	3580	Reshui	17.8	100.4719 °E, 37.5086° N
EP26	Female	190	3580	Reshui	17.8	100.4719 °E, 37.5086° N
EP27	Male	186	3580	Reshui	17.8	100.4719 °E, 37.5086° N
EP28	Female	147	3580	Reshui	17.8	100.4719 °E, 37.5086° N
EP29	Female	211	3580	Reshui	17.8	100.4719 °E, 37.5086° N
EP30	Male	157	3580	Reshui	17.8	100.4719 °E, 37.5086° N
EP31	Female	146	3580	Reshui	17.8	100.4719 °E, 37.5086° N
EP32	Male	161	3580	Reshui	17.8	100.4719 °E, 37.5086° N
EP33	Female	86	3580	Reshui	17.8	100.4719 °E, 37.5086° N
EP34	Female	52	3580	Reshui	17.8	100.4719 °E, 37.5086° N
EP35	Male	146	3694	Xiaderi	17.7	101.4429°E, 35.0189° N

EP36	Female	157	3694	Xiaderi	17.7	101.4429°E,	35.0189° N
EP37	Male	166	3694	Xiaderi	17.7	101.4429°E,	35.0189° N
EP38	Male	153	3694	Xiaderi	17.7	101.4429°E,	35.0189° N
EP39	Female	150	3694	Xiaderi	17.7	101.4429°E,	35.0189° N
EP40	Female	148	3694	Xiaderi	17.7	101.4429°E,	35.0189° N
EP41	Male	156	3694	Xiaderi	17.7	101.4429°E,	35.0189° N
EP42	Female	133	3694	Xiaderi	17.7	101.4429°E,	35.0189° N
EP43	Male	188	3694	Xiaderi	17.7	101.4429°E,	35.0189° N
EP44	Male	160	3694	Xiaderi	17.7	101.4429°E,	35.0189° N
EP45	Male	160	3694	Xiaderi	17.7	101.4429°E,	35.0189° N
EP46	Female	148	3694	Xiaderi	17.7	101.4429°E,	35.0189° N
EP47	Male	184	3694	Xiaderi	17.7	101.4429°E,	35.0189° N
EP48	Male	156	3694	Xiaderi	17.7	101.4429°E,	35.0189° N
EP49	Female	148	3694	Xiaderi	17.7	101.4429°E,	35.0189° N
EP50	Female	168	3694	Xiaderi	17.7	101.4429°E,	35.0189° N
EP51	Female	96	3694	Xiaderi	17.7	101.4429°E,	35.0189° N
EP52	Female	133	3856	Wangjiaxiang	16.2	101.4605°E,	35.7363° N
EP53	Female	123	3856	Wangjiaxiang	16.2	101.4605°E,	35.7363° N
EP54	Male	167	3856	Wangjiaxiang	16.2	101.4605°E,	35.7363° N
EP55	Female	130	3856	Wangjiaxiang	16.2	101.4605°E,	35.7363° N
EP56	Male	134	3856	Wangjiaxiang	16.2	101.4605°E,	35.7363° N
EP57	Male	182	3856	Wangjiaxiang	16.2	101.4605°E,	35.7363° N
EP58	Female	146	3856	Wangjiaxiang	16.2	101.4605°E,	35.7363° N
EP59	Male	168	3856	Wangjiaxiang	16.2	101.4605°E,	35.7363° N
EP60	Female	110	3856	Wangjiaxiang	16.2	101.4605°E,	35.7363° N
EP61	Male	172	3856	Wangjiaxiang	16.2	101.4605°E,	35.7363° N
EP62	Male	113	3856	Wangjiaxiang	16.2	101.4605°E,	35.7363° N
EP63	Female	136	3856	Wangjiaxiang	16.2	101.4605°E,	35.7363° N
EP64	Male	166	3856	Wangjiaxiang	16.2	101.4605°E,	35.7363° N
EP65	Female	155	3856	Wangjiaxiang	16.2	101.4605°E,	35.7363° N
EP66	Male	174	3856	Wangjiaxiang	16.2	101.4605°E,	35.7363° N
EP67	Male	166	3856	Wangjiaxiang	16.2	101.4605°E,	35.7363° N
EP68	Female	156	3856	Wangjiaxiang	16.2	101.4605°E,	35.7363° N
EP69	Female	216	4331	Guoluo	14.3	100.1297°E,	34.1411°N
EP70	Female	132	4331	Guoluo	14.3	100.1297°E,	34.1411°N
EP71	Female	178	4331	Guoluo	14.3	100.1297°E,	34.1411°N
EP72	Male	127	4331	Guoluo	14.3	100.1297°E,	34.1411°N

EP73	Female	196	4331	Guoluo	14.3	100.1297°E, 34.1411°N
EP74	Male	72	4331	Guoluo	14.3	100.1297°E, 34.1411°N
EP75	Female	68	4331	Guoluo	14.3	100.1297°E, 34.1411°N
EP76	Male	103	4331	Guoluo	14.3	100.1297°E, 34.1411°N
EP77	Male	169	4331	Guoluo	14.3	100.1297°E, 34.1411°N
EP78	Male	187	4331	Guoluo	14.3	100.1297°E, 34.1411°N
EP79	Male	158	4331	Guoluo	14.3	100.1297°E, 34.1411°N
EP80	Female	65	4331	Guoluo	14.3	100.1297°E, 34.1411°N
EP81	Female	173	4331	Guoluo	14.3	100.1297°E, 34.1411°N
EP82	Male	186	4331	Guoluo	14.3	100.1297°E, 34.1411°N
EP83	Male	152	4331	Guoluo	14.3	100.1297°E, 34.1411°N
EP84	Male	135	4331	Guoluo	14.3	100.1297°E, 34.1411°N
EP85	Female	133	4331	Guoluo	14.3	100.1297°E, 34.1411°N

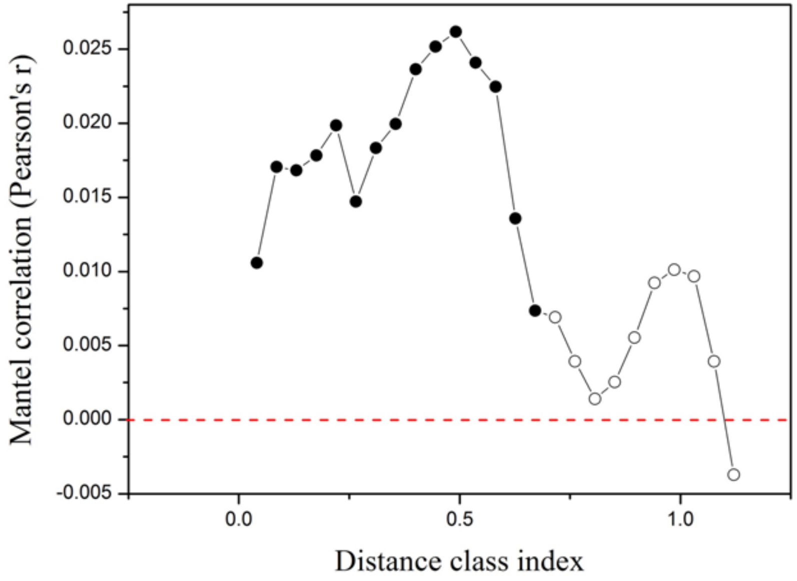

Fig. S1 Mantel correlation between the pairwise matrix of OTU niche distances and the phylogenetic distances in pika gut microbiota with 999 permutations. Significant correlations ($P < 0.05$) of phylogenetic signals in species ecological niches are marked as solid circles, whereas non-significant correlations are labeled as hollow circles.

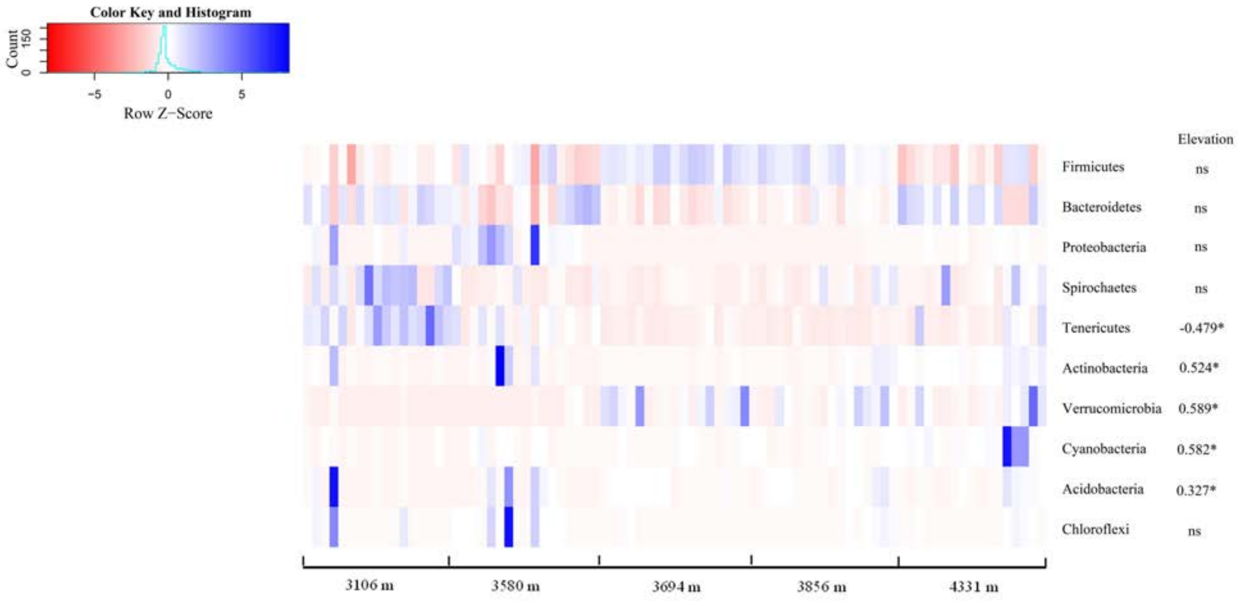

Fig. S2 The abundance distribution of major phyla in pika gut across elevations and their corrlation with elevation. The relative abundance of these phyla was normalized using Z-score transformation Only those phyla with mean relative abundance > 0.05% across all samples are shown. Spearman correlation analysis was used to detect the correlation between the relative abudance of these genera and elevation. The symbol (*) means significant correlations with $P < 0.05$. NS signifies no significance.

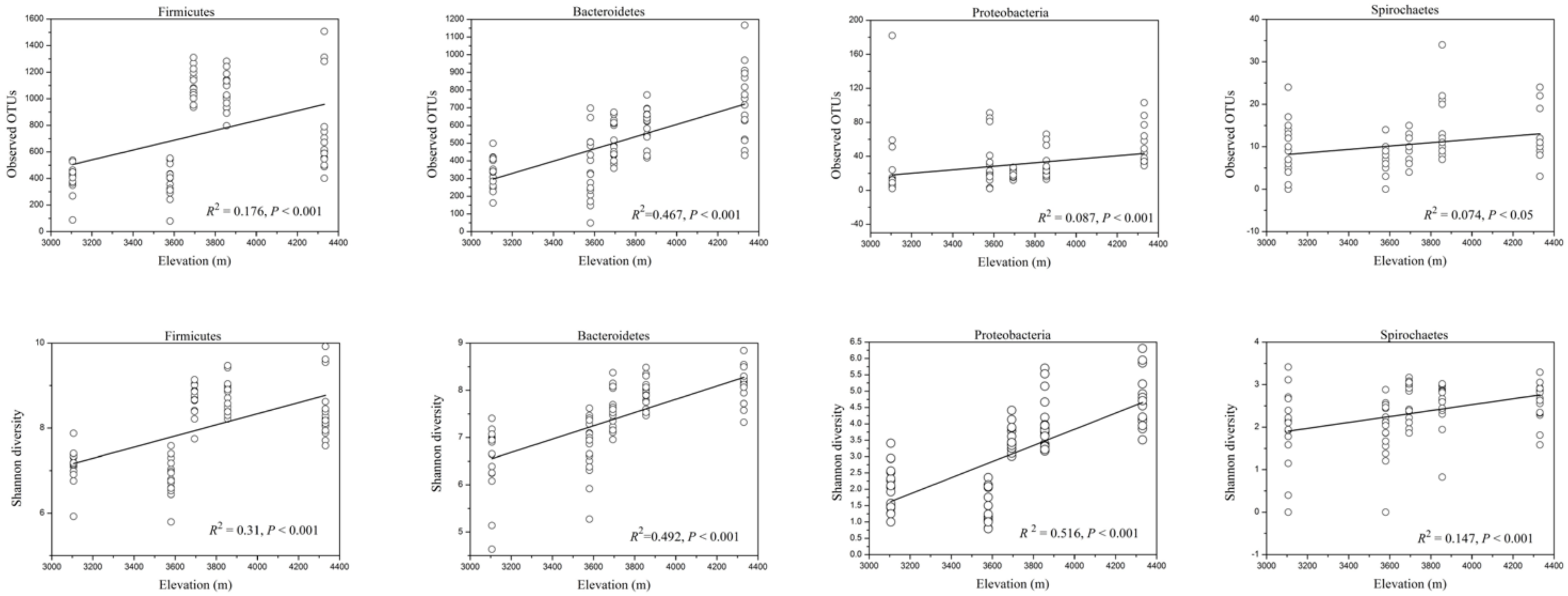

Fig. S3 Alpha diversity (observed OTUs and Shannon diversity) values of the dominant phyla Firmicutes, Bacteroidetes, Proteobacteria and Spirochaetes were significantly correlated with elevation (All P values < 0.05).

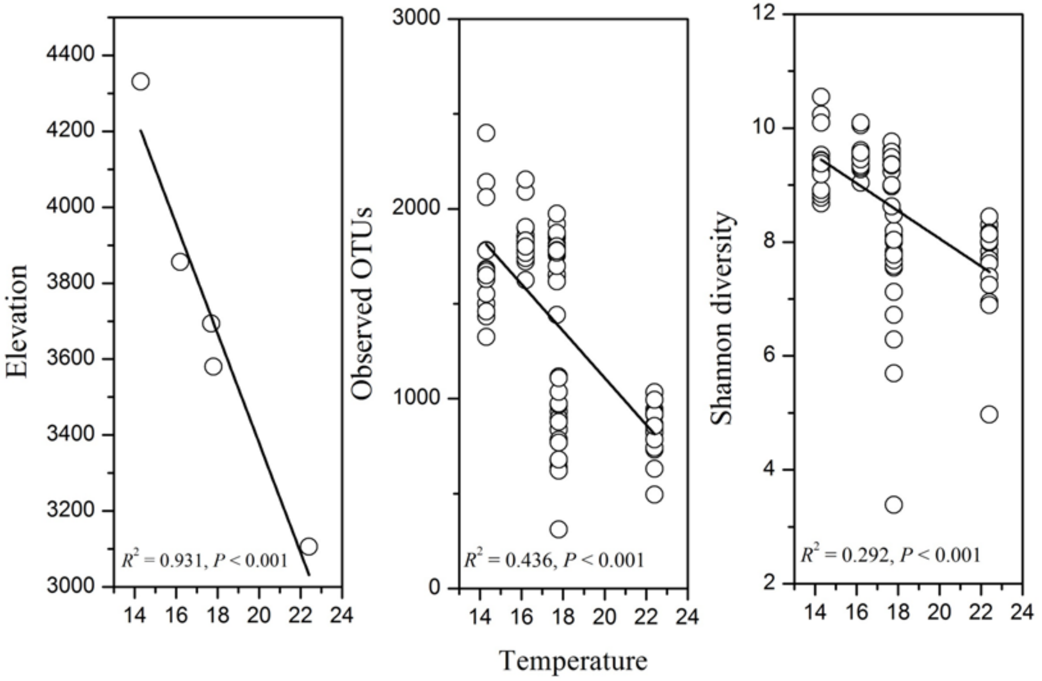

Fig. S4 The linear regression rela tionship between temperature and elevation, observed OTUs or Shannon diversity.

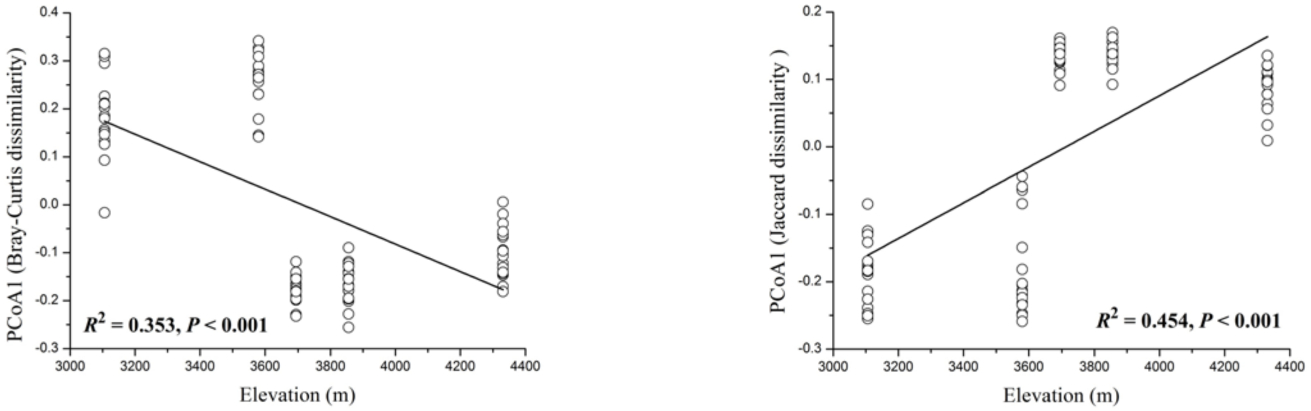

Fig. S5. Relationship between elevation and principal coordinate axis 1(PCoA1) from Jaccard and Bray-Curtis dissimilarities for pika gut microbiota. PCoA1 is significantly correlated with Jaccard or Bray-Curtis distance.

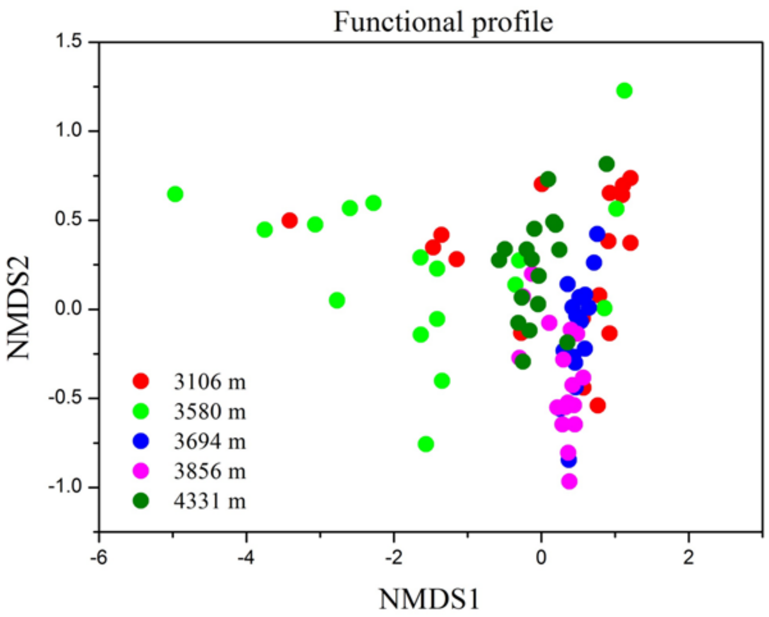

Fig. S6 Non-metric multidimensional scaling (NMDS) plots showing the difference of gene functional profiles acorss elevations at level 3 based on Bray-Curtis distance.

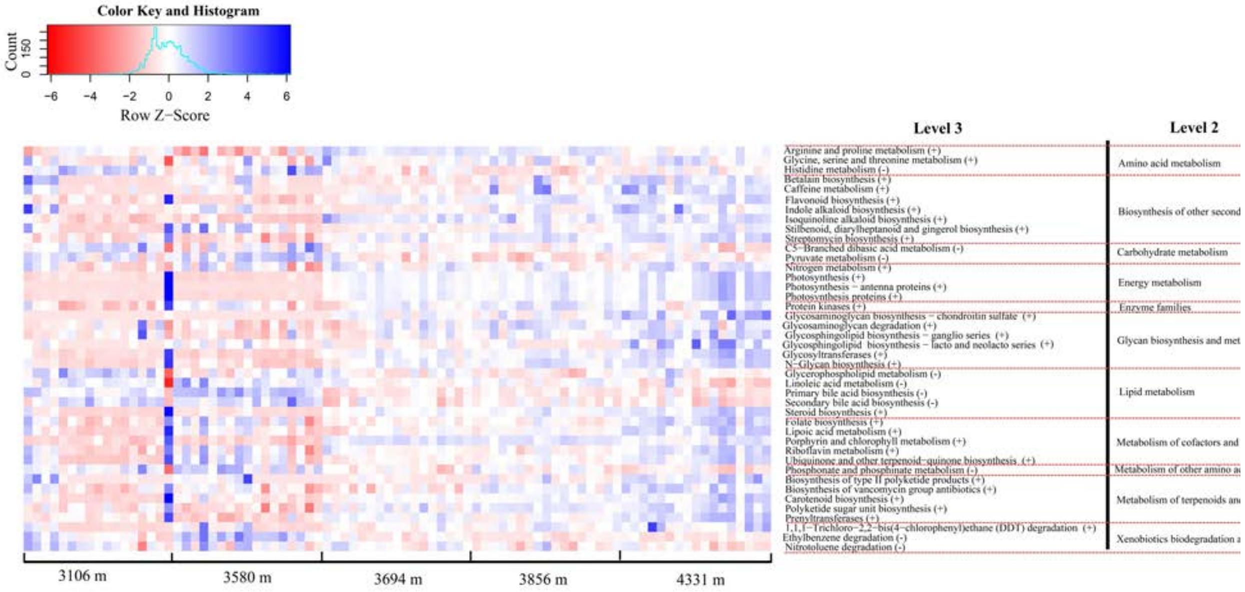

Fig. S7 The distribution of the predicted gene functions associated with metabolism at level 2 across elevations. The relative abundance of gene functions was normalized using Z-score transformation. Only those gene functions that correlated with elevation ($r > 0.3$ or < -0.3, $P < 0.01$) are shown (all P values < 0.001).

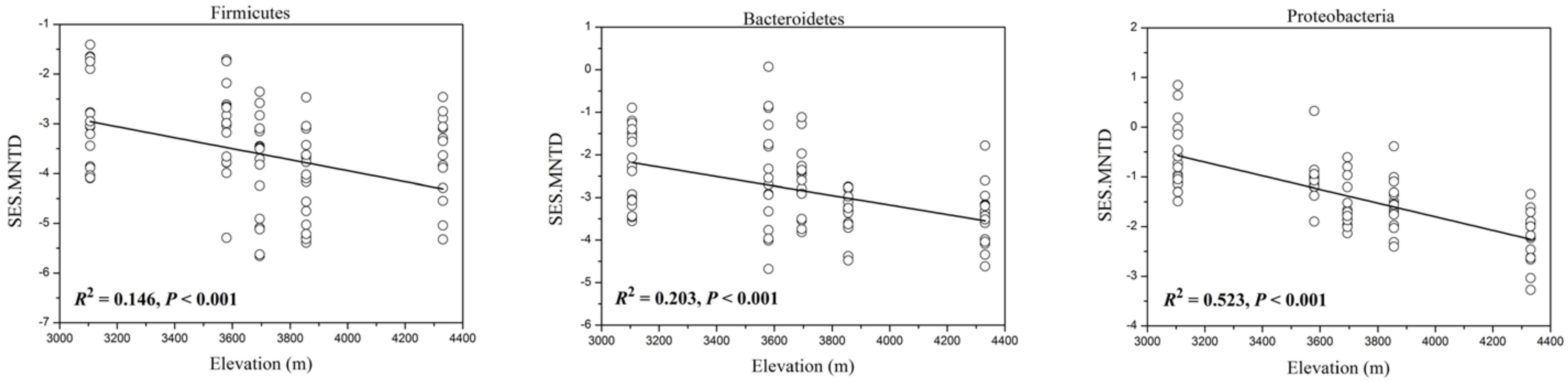

Fig. S8 The weighted standardized effect size of the mean nearest taxon distance (SES.MNTD) for the three dominant phyla Firmicutes, Bacteroidetes, Proteobacteria was significantly correlated with elevation (all *P* values < 0.001).

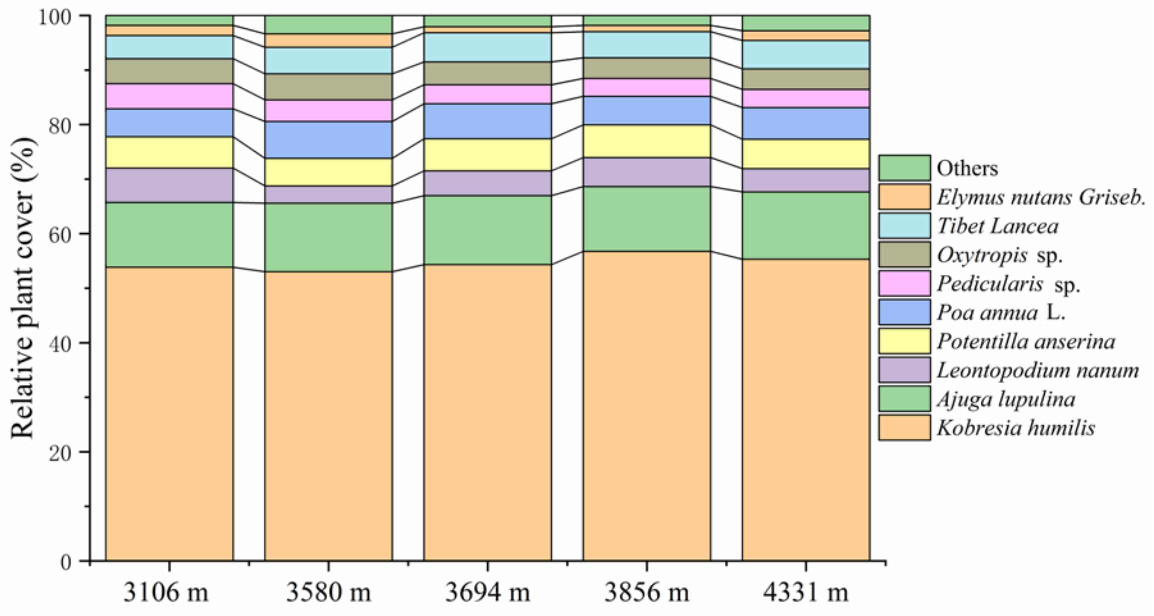

Fig. S9 The composition of plant communities in each elevation. Only nine most abundant plant species were shown.
